# Rapid, Precise and Affordable Estimation of Venlafaxine and Its Metabolites in Highly Polluted Effluent Waters: Proof-of-Concept for Methodology

**DOI:** 10.3390/molecules25204793

**Published:** 2020-10-19

**Authors:** Snawar Hussain, Chandramouli Ramnarayanan, Teeka S. Roopashree, Md. Khalid Anwer, Nagaraja Sreeharsha, Anroop B. Nair

**Affiliations:** 1 Department of Biomedical Sciences, College of Clinical Pharmacy, King Faisal University, Al-Ahsa 31982, Saudi Arabia; 2 Department of Quality Assurance, Krupanidhi College of Pharmacy, Bengaluru 560035, India; pharmwhiz@gmail.com; 3 Department of Pharmacognosy, Government College of Pharmacy: Bangalore, Karnataka 560027, India; ts.roopa@gmail.com; 4 Department of Pharmaceutics, College of Pharmacy Prince Sattam Bin Abdulaziz University, Al-Alkharj 11942, Saudi Arabia; m.anwer@psau.edu.sa; 5 Department of Pharmaceutical Sciences, College of Clinical Pharmacy, King Faisal University, Al-Ahsa 31982, Saudi Arabia; anair@kfu.edu.sa; 6 Department of Pharmaceutics, Vidya Siri College of Pharmacy, Off Sarjapura Road, Bangaluru, Karnataka 560035, India

**Keywords:** venlafaxine metabolites, spectrophotometry, antidepressant, venlafaxine, water pollution, environmental samples, wastewater treatment

## Abstract

Widespread presence of pharmaceuticals and their metabolites in the environment of industrialized countries is an emerging global concern. Potential contamination of the soil and water by such pharmacologically active substances poses serious ecotoxicological implications. Several studies assessing the long-term ecological risks of pharmaceutical contaminants mainly focus on the risk assessment of the parent drug, while the potential contributions of their metabolites is often neglected. Presence of selective serotonin and norepinephrine reuptake inhibitor venlafaxine, an antidepressant drug, and its metabolites is a matter of serious concern for aquatic systems, since they are difficult to remove by traditional wastewater treatment processes. The concentration of VEN present in water is reportedly one of the highest among pharmaceuticals; however, the long-term effects of its metabolites have not yet been systematically studied. Given the consideration to complex and time-consuming effluent treatment, and realizing the importance of levels of venlafaxine and its metabolites, a simple and accurate analytical method for quick determination is needed. We designed a selective colorimetric method by using oxidative coupling of drug molecules with 3-methyl-2-benzothiazolinone hydrazone hydrochloride (MBTH) reagent, to quantify the presence of venlafaxine and its metabolites in aquatic samples, with special emphasis on effluent. The method was validated for selectivity, specificity and robustness as per the ICH Q2 guidelines to assess its suitability in pharmaceutical samples, as well. Highly sensitive and green economical analytical method was successfully established for estimation of venlafaxine and its metabolites in aquatic samples. The method was quick, as it involved minimum processing steps. The method was accurate and linear in the range of 0.5 to 80 ppm and could successfully detect lowest concentration of 1.3 ppm, thus qualifying its applicability for the desired purpose to check the presence of trace levels of VEN or its metabolites in aquatic samples or in pharmaceutical formulations.

## 1. Introduction

A comprehensive understanding of the potential environmental micro-pollution caused by pharmaceutical drugs and their metabolites in aquatic or terrestrial ecosystems has become a cause of global concern in recent years. The pharmaceutical active ingredients and their biotransformed metabolites are increasingly found to contaminate the aquatic systems through sewage and effluents, since they are readily excreted out of the human body, through feces and/or urine. A study conducted at the Harvard Medical School demonstrated that an average US household has a sizable stock of unused and expired medicines, and a majority of these are not disposed of properly [1]. The poor disposal practices followed by small hospitals and nursing clinics involve flushing off expired drugs, thus also contributing to the increased pharmaceutical load in the wastewater. The traditional wastewater-treatment techniques currently employed do not remove these pharmaceutical compounds completely [2].

Furthermore, many livestock industries use antibiotics and drug formulations, and their effluent run-offs often contaminate surface water. The pharmaceutical manufacturing industries also contribute to the downstream pollution, since their effluent often contains active pharmaceutical ingredients, as well as their by-products.

The harmful effects of pharmaceutical agents on the aquatic ecosystems, especially in the context of the health risk imposed on fish, have been documented [3,4]. A qualitative study, as reported by M. Schultz and his team, focused on detecting the presence of antidepressants in effluent-impacted water bodies detected a maximum abundance of antidepressant venlafaxine (VEN), in comparison to other antidepressant drugs like fluoxetine, bupropion, etc. [4]. The presence of VEN in many effluent-impacted downstream waters has been documented previously [5,6,7], and its concentration was found to be much higher than the other drugs [4,8,9,10]. VEN increased mortality in exposed embryos and also affected the reproduction, along with significant histological changes in the kidney of female zebrafish [11]. Integration of vibrational, auditory and visual information required for predator avoidance response namely C-start strategy was significantly reduced in *Pimephales promelas* after embryonic and larval exposure to VEN [3]. The considerable bio-magnification of these pharmaceuticals and their potentially active metabolites in the top members of the food chain result in redistribution within the biosphere, making them persistent organic pollutants.

**Figure 1:**
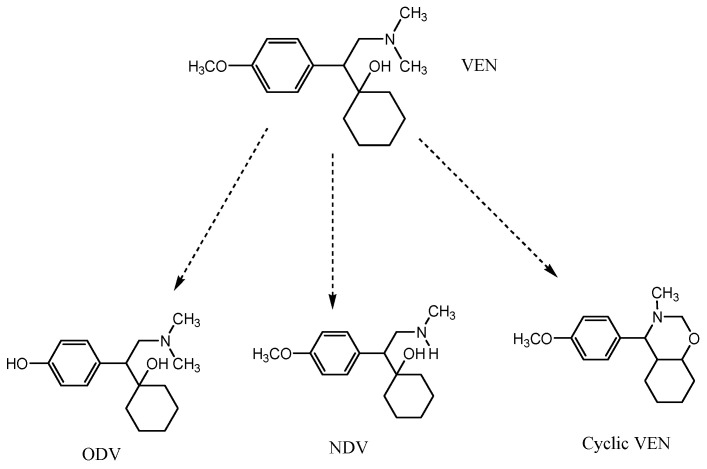
Metabolic reactions of venlafaxine (VEN) to produce O-Desmethylvenlafaxine (ODV), N-Desmethylvenlafaxine (NDN) and Cyclic VEN.

VEN, a frequently prescribed antidepressant, undergoes extensive metabolism following oral administration forming metabolites, viz, Cyclic VEN, O-Desmethylvenlafaxine (ODV) and N-Desmethylvenlafaxine (NDV) (Figure 1) [12]. Given the consideration to the risk of bioaccumulation and danger to the aquatic life, control over levels of these chemicals in wastewater need to be established. Hence, a reliable method to accurately detect and quantify VEN and its metabolites is essential. Due to the extensive metabolism of VEN in the liver and the gut, its bioavailability is reportedly low [13]. Both ODV and NDV eventually get metabolized into *N,N*,*O*-tridesmethyl venlafaxine and are excreted as glucuronide complexes. An alternative metabolic pathway of VEN involves direct inter-molecular cyclization to produce another metabolite 5-(4-Methoxyphenyl)-3-methyl-1-oxa-3-azaspiro [5] undecane (Cyclic VEN) [14]. Among these metabolites, ODV is the main pharmacologically active metabolite having a similar mechanism of action like the parent drug, VEN [15]. The presence of other minor metabolites in the body fluids and their moderate inhibitory activity towards serotonin and nor-epinephrine enzymes is also reported [13]. The presence of these metabolites, along with VEN, in the sewage adversely affects the life of aquatic species due to their significant biological effects. The microbial or environmental controlled degradation of VEN may also produce structurally similar degradation products in downstream waters. The enantio-specific toxicity and bioaccumulation VEN and ODV is studied and reported earlier [16]. In another study, VEN showed reasonable resistance towards both microbial and light-assisted degradation, while its desmethyl metabolites readily underwent stereo-selective degradation [17]. The stereochemistry of a compound is known to influence its degradation pathways. In particular, chiral enantiomers have the potential to interact with a biologically mediated environment, resulting in the enrichment of selected enantiomer/s in water [18,19]. VEN and its metabolites, being chiral compounds, are expected to undergo stereo-selective degradation in downstream waters, as well as activated-sludge-treated waters.

The fate and the long-term effects of these chiral metabolites of antidepressants are not yet explored systematically.

The amounts of pharmaceuticals and their subsequent degradation products found in surface waters are generally trace level and in the range of 0.2 to 3 ng/L, due to the mixing up of these chemicals with a large volume of water [20,21]. Many times, the samples are collected at 8 to 10 km downstream, away from the suspected sources [4]. This calls for ultra-trace level detection techniques such as UPLC and capillary electrophoresis with electrospray ionization mass spectrometry [20,21]. Though these techniques are essentially sensitive for the trace-level detection, they require complicated and expensive instrumentation setups, high levels of operational skills and considerable time for analysis and, hence, are not suitable for quick and routine analysis of water samples.

In addition, many underdeveloped countries do not follow systematic treatment of effluent water, and rampant downstream pollution is observed in water bodies close to pharmaceutical industries. The extent of pharmaceutical pollution in such countries is roughly 150 times more than that in the United States [22,23,24,25,26,27,28]. The water pollution in India by pharmaceuticals has now emerged as a major concern because of extensive industrialization of manufacturing hub with 300 large-scale and 8000 medium-scale production units [29,30]. In developing country, effluent treatment procedures are still not rigorously followed, and many production units dispose of the effluents straight to the water bodies [31,32,33]. In one of the case studies conducted in a pharmaceutical manufacturing hub near Hyderabad, India, the presence of certain broad-spectrum drugs was found to be as high as 31 µg/mL in water samples [34]. In another study, the presence of pharmaceuticals was found to be as high as 14 µg/mL in effluents and 6.5 µg/mL in lake water near industrial area in Hyderabad [35]. All of these studies clearly indicate significantly high levels of pharmaceuticals at the effluent stage as well, as in the environment. This in-turn reflects the poor state of affairs of improper effluent treatment practices prevalent in many countries. VEN and its metabolites were not part of the above-described studies, and, therefore, we have selected them, considering the high probabilities of occurrence of these ecotoxic molecules in effluent water and their potential harmful environmental impacts [36].

Therefore, it is important to develop and optimize a robust, reliable and specific detection method to analyze the presence of VEN and its metabolites in effluent water systems and also in surface water near highly polluted industrial areas. We developed a simple, reproducible, easy and highly convenient spectrophotometric method to detect and quantify the VEN and its metabolites in the water samples. The salient features of this method include minimum processing steps, the absence of secondary extraction steps or use carcinogenic solvents, and its robustness against excipients usually present in the aquatic environmental samples. The developed method was validated by using the International Conference on Harmonization (ICH) Q2 guidelines [36], and potential applicability to detect and quantify VEN and VEN metabolites in pharmaceutical formulations was also tested, to expand the scope of method for future application to body samples.

## 2. Results and Discussion

### 2.1. Proposed Reaction Mechanism

**Figure 2:**
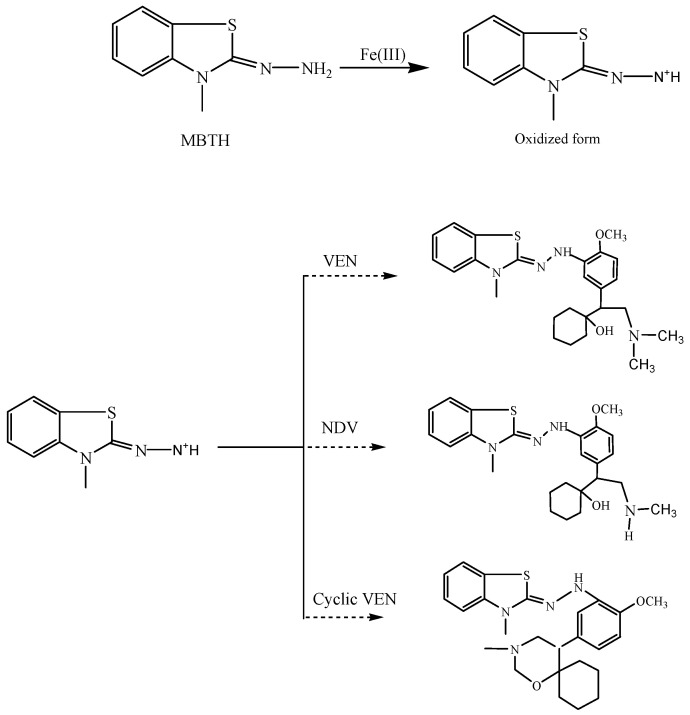
Proposed reaction mechanism/s producing the spectrophotometrically detectable colored complexes.

The proposed reaction mechanism involves oxidation of MBTH into an electrophilic intermediate on treatment with Ferric Chloride (III). This electrophilic intermediate then undergoes complexation with VEN and its intermediates, to produce bluish-green-colored complexes (Figure 2).

**Figure 3:**
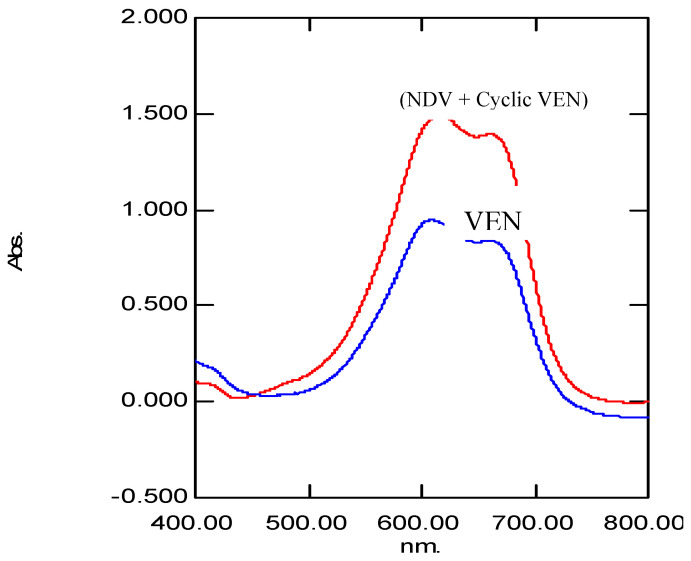
UV–Vis spectra showing absorption maxima of colored complex of VEN (608.5 nm) and colored complex of NDV + Cyclic VEN (616.5 nm).

Under favorable reaction conditions, MBTH undergoes oxidation to form an electrophilic intermediate, which gets stabilized by resonance. VEN and its metabolites undergo complexation with the oxidized form of MBTH, to form colored complexes which showed absorption maxima at 608.5 and 616.5 nm, respectively (Figure 3).

**Figure 4:**
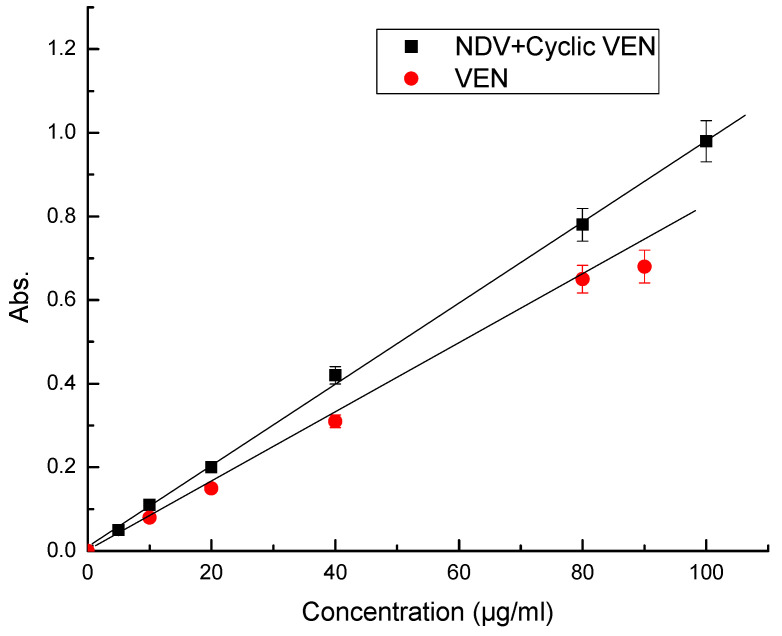
Calibration curves of colored complexes of VEN and mixture of NDV + Cyclic VEN.

Optimized reaction conditions and a series of six standard solutions were used to construct the calibration plot. The optical densities were found to increase linearly with the concentration of the analyte (Figure 4), in the concentration range of 0.5–80 and 0.5–100 µg/mL for VEN and the mixture of NDV + Cyclic VEN, respectively. Other parameters, such as the molar extinction coefficient and the Sandell’s sensitivity, were obtained from the Beer Lambert’s plot. A relatively high molar extinction coefficient further underlines the sensitivity of the developed method. The plot showed a relatively small intercept value as obtained by using the equation of least squares, as mentioned below (Equation (1)):

A = a + bc,(1)

where ‘A’ is the absorbance of the sample taken in a cell of path length 1 cm, ‘a’ is the intercept, ‘b’ is the slope and ‘c’ is the concentration in µg/mL.

Accordingly, the concentration of an unknown solution of the analytes may be calculated from the calibration plot, as shown below (Equation (2)):

Unknown Absorbance = Intercept+ (Slope × Concentration),(2)

The values of the limit of detection and limit of quantification were determined by using the equations below (Equations (3) and (4)):

(3) $$LoD\;=\frac{3.3S_{B}}{a}$$

(4) $$LoQ\;=\frac{10S_{Q}}{a}$$

where *S_B_* = standard deviation of six replicate reagent blank determinations, and *a* = slope of the calibration plot.

The regression plots were also derived, and the regression parameters are listed in Table 1.

### 2.2. Reaction Stoichiometry

**Figure 5:**
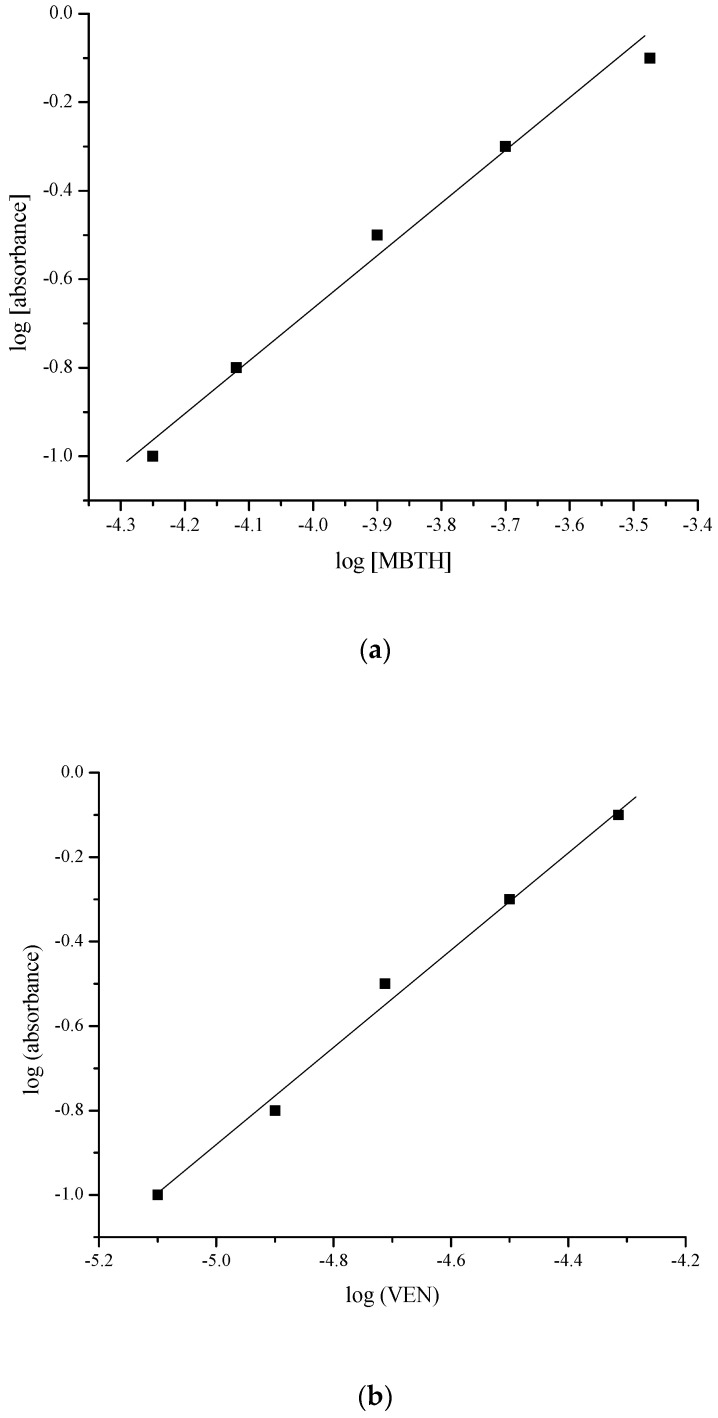
Limiting logarithmic plot: (**a**) log [absorbance] vs. log [concentration of 3-methyl-2-benzothiazolinone hydrazone hydrochloride (MBTH)]; (**b**) log [absorbance] vs. log [concentration of VEN].

As evident from the reaction mechanism, the coupling occurs at one of the ortho positions of the drug molecules, and the stoichiometry of the reaction has been confirmed by the limiting logarithmic method [37]. The plots of log [absorbance] vs. log [concentration of MBTH], and log [absorbance] vs. log [concentration VEN] yielded linear relationships with slopes of 1.159 and 1.162, respectively (Figure 5). This clearly demonstrated that the stoichiometry of the reaction was 1:1.

### 2.3. Effects of Reagent Concentration

**Figure 6:**
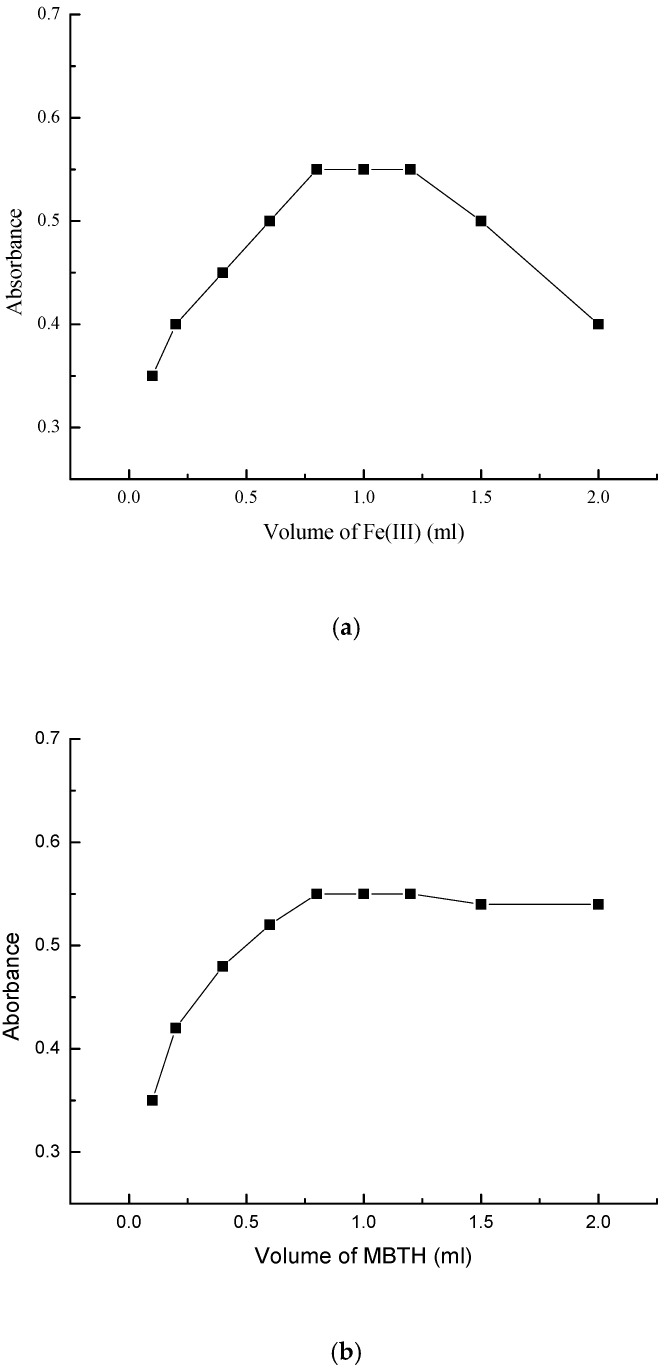
Effect of concentration of (**a**) Fe (III) and (**b**) MBTH.

The concentrations of MBTH and Fe (III) solutions used were found to have an effect on the absorption maximum. For detectable absorption values, the optimal concentration of MBTH was found to be 1.0 mL of 0.08% solution. An excess of MBTH leads to decreased optical density and elevated color intensity of the blank, due to the presence of unreacted MBTH [38]. Similarly, the ideal amount of Fe (III) solution was found to be 1.0 mL of 0.05% solution, and, beyond this level, there are chances of oxidation of the complexation product. The effects of concentrations of MBTH and Fe (III) on the absorbance are illustrated below, in Figure 6a,b.

### 2.4. Method Validation

The statistical validation of the reported method was performed as per the ICH Q2 (R1) guidelines. The parameters, such as precision and accuracy, were established by carrying out intra-day and inter-day recovery studies. This was performed by following the standard addition technique, in which a known amount of the pure reactant drug was added to the pre-analyzed dose. The net amount of the analyte after the addition was measured by using the proposed procedure. For the intra-day measurements, the relative standard deviations were found to range between 0.5029 and 2.9141, and between 0.5020 and 2.8994 for VEN and [NDV + Cyclic VEN] samples, respectively (Table 2).

### 2.5. Interference Study

Spectrophotometric methods often suffer from interference, due to commonly used excipients in the formulations. The proposed method is resistant to any such interference, as observed from the recovery study results (Table 3).

The recovery studies showed that the sensitivity of the proposed spectrophotometric method does not suffer from interference from commonly used formulation excipients of varied aqueous solubility characteristics like highly hydrophobic talc and highly hydrophilic dextrose. Concentration of excipients selected was 10 times the concentration of drug, except for starch and cellulose. The method also shows specificity, as analysis was not affected even in presence of starch and cellulose, which were 15 and 20 times the drug concentration.

The developed method was also tested for the effect of commonly used surfactants frequently found in effluent water, and the results are listed in Table 4.

### 2.6. Effect of Adding Metabolites into VEN Tablet Samples

It was found that adding the metabolites NDV and Cyclic VEN to the VEN tablet solution increases the color intensity and subsequently the value of λ_max_. An amount as small as 0.5 µg/mL (of the metabolites) was enough to alter the absorbance value. The resultant absorbance showed a linear increase with the concentration of metabolites added (Table 5).

### 2.7. Kinetic Studies

**Figure 7:**
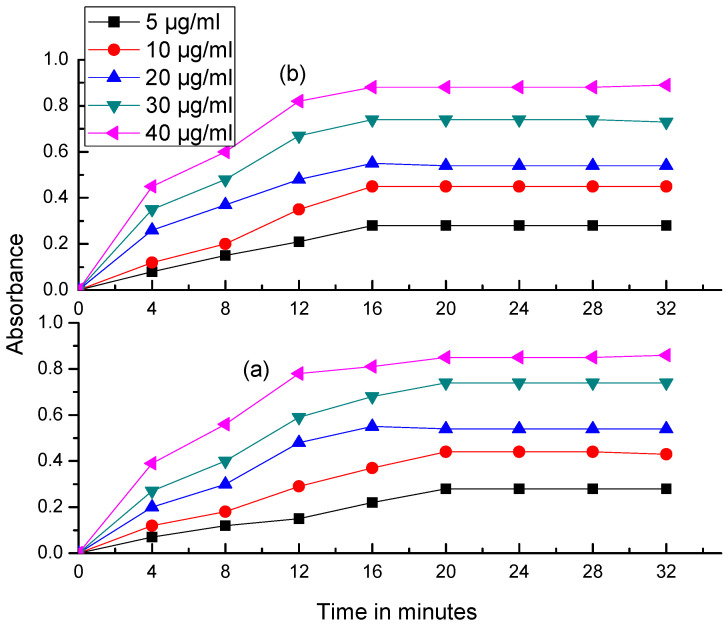
Absorbance vs. time plots: (**a**) VEN and (**b**) [NDV + Cyclic VEN].

For the quantitative determination of VEN and its metabolites under the optimal reaction conditions, pseudo-order reaction kinetics is expected, as the concentration of Fe (III) is constant. This means that the rate of the complexation reaction primarily depends on the concentration of VEN as rate = k’ [VEN]. For the rate studies, initial rate and fixed absorbance methods were found to be unsuitable, and, hence, the fixed time method was employed for getting the desired sensitivity and regression parameters. For the fixed time method, the absorbance of samples containing 5, 10, 20, 30 and 40 µg/mL of VEN and [NDV + Cyclic VEN] were measured after fixed time intervals of 4, 8, 12, 16, 20, 24, 28 and 32 min, and a graph was plotted with absorbance against time (Figure 7). It was noted that the slope of the calibration plots increased with time, and the optimal regression parameters are obtained at 20 min for VEN and at 16 min for [NDV + Cyclic VEN]. Therefore, it can be concluded that the λ_max_ measurement should be out after the reaction time of 20 and 16 min for VEN and [NDV + Cyclic VEN], respectively, to get stable, consistent and reproducible results.

## 3. Materials and Methods

### 3.1. Materials

VEN, Cyclic VEN and NDV were received as gift samples from M/s Microlabs Ltd., India, and were used without any processing. All reagents used were of AR grade, and double-distilled water was used for making all the dilutions.

Stock solution: The 50 µg/mL stock solutions of VEN, Cyclic VEN and NDV were prepared by dissolving their respective amounts in 1 mL methanol, followed by dilution with an appropriate amount of distilled water.

Ferric Chloride, 0.05% (*w/v*) aqueous solution; 3-Methyl-2-benzothiazolinone hydrazone hydrochloride (MBTH), 0.08% (*w*/*v*) aqueous solution.

### 3.2. Instrumental

A UV–Visible spectrophotometer (UV-2600 Shimadzu, Japan) with matched quartz cuvettes was used for all spectroscopic measurements.

### 3.3. Method Development

MBTH was utilized previously to estimate drug molecules containing phenolic and amino groups [39]. There have been limited efforts in developing and optimizing spectrophotometric methods to date for the estimation of VEN metabolites, such as NDV and Cyclic VEN.

MBTH undergoes an oxidative coupling reaction, with the molecules of interest being estimated to produce colored complexes. The colored end product of the oxidative coupling reaction was further quantified by using a UV/Vis spectrophotometric method at the absorption maximum.

### 3.4. Optimization of Reaction Conditions and Procedures

The stoichiometry of the reaction was studied by adopting the limiting logarithmic method [37]. Two straight lines were obtained upon using increasing concentrations of MBTH while keeping the concentration of VEN constant and also upon using increasing concentrations of VEN, keeping the concentration of the MBTH constant. The highest absorption maximum was obtained when the following concentrations of the reagents were added in the sequence mentioned below:

#### 3.4.1. VEN 

A series of aliquots containing 0.5 to 10 µg/mL of VEN were taken into 10 mL standard volumetric flasks. To this, 1.0 mL of freshly prepared ferric chloride solution was added, followed by the addition of 1.0 mL freshly prepared MBTH solution. The solution was mixed thoroughly, and the volume was adjusted with water. A bluish-green-colored solution was obtained and was scanned between 400 to 800 nm, against a reagent blank on UV–Vis spectrophotometer.

#### 3.4.2. NDV and Cyclic VEN

A series of aliquots containing the 0.5 to 10 µg/mL of NDV and Cyclic VEN (in 50:50 ratio) were taken into a 10 mL standard flask. To this, 1.0 mL of freshly prepared ferric chloride solution was added, followed by the addition of 1.0 mL freshly prepared MBTH solution. The solution was mixed thoroughly, and volume was adjusted with water. The bluish-green-colored solution obtained was scanned between 400 to 800 nm, against a reagent blank, similar to the procedure followed for VEN.

### 3.5. Calibration Curve

A series of aliquots containing VEN and a mixture of NDV and Cyclic VEN, containing 0.5 to 100 µg/mL each, were transferred into 10 mL standard flasks. The colored complexes were formed by following the steps listed above. The optical density values obtained were plotted against the concentration of the analyte (µg/mL), to get a calibration curve. A regression analysis was also carried out with the set of absorbance values, to determine the best-fit equation.

### 3.6. Procedure for VEN Tablets (Recovery Studies)

Locally available over-the-counter tablets (10 in number) of VEN 37.5 mg (Venlor, Cipla Ltd., India) and Venfax PR37.5 mg (Microlabs, India) were powdered to form a homogeneous sample. A representative sample equivalent of 5 mg of VEN was transferred into a 25 mL volumetric flask. Methanol 1 mL was added to disperse the constituents, and the volume was adjusted with water. This solution was filtered through a Whatman 40 filter paper, and the filtrate was diluted suitably before analyzing spectrophotometrically, as per the procedure discussed above.

### 3.7. Effects of Surfactants and Excipients

As aquatic samples usually contain commonly used surfactants, the influence of the same on the proposed method and its application was also studied. Aliquots containing 10 µg/mL of VEN were transferred into a standard volumetric flask, and known amounts of commonly used surfactants such as sodium lauryl sulfate, gelatin and sodium stearate were added prior to spectrophotometric estimation. Tablets, as well as the other pharmaceutical dosage forms, contain a variety of excipients, and their presence may potentially influence the analytical method. In order to verify the robustness of the developed method, known amounts of commonly used excipients such as starch, talc, gum, dextrose, alginate, cellulose, etc., were added into aliquots containing 10 µg/mL of VEN. The resultant solutions were analyzed spectrophotometrically by using freshly prepared reagent blank.

The variation in the absorbance values after adding the surfactants and excipients were analyzed and interpreted as per the ICH Q2 guidelines.

### 3.8. Standard Addition of Metabolites into VEN Tablet Solution

Finely powdered tablet samples were spiked with varying amounts of NDV and Cyclic VEN, and the corresponding changes in the absorbance were noted. Spectrophotometric measurements were performed as per the general procedure specified above.

## 4. Conclusions

Although spectrophotometric methods are reported in the literature for the estimation of VEN, this study is seminal in reporting a reliable, accurate and precise method for the determination of VEN metabolites NDV and Cyclic VEN. The method reported in this study appeared to be robust, since it is pH independent and remains unaffected by solvent conditions. The colored complexes formed were found to be stable up to 3 h, and they showed no signs of decomposition when exposed to light. The method is found to be easy, straightforward, inexpensive, reproducible and accurate, and it does not involve any cumbersome reaction conditions or extraction steps. It did not suffer interference from surfactants or excipients, making it ideal for aquatic samples from various ecosystems. The method was found to be linear over a broad concentration range that may occur in industrial effluent samples.

Though the reported method was not sensitive enough to test the ultra-trace-level pharmaceutical concentrations in the vast surface waters, it can be used for the routine detection of higher concentrations in high-risk areas, as noticed frequently in the industrial-waste discharge. The method can be easily employed for routine quality control tests in effluent waters and lake or well samples in and around highly polluted pharmaceutical industrial areas. Even though the severe water contamination in underdeveloped countries is of concern to the local areas, bioaccumulation of toxic chemicals in aquatic life results in recirculation in the biosphere, eventually causing hazard to the entire ecosystem. Eventually, it highlights the moral responsibility of pharmaceutical manufacturers from both underdeveloped and developed countries.

This method may also find a scope for applications in the detection and quantification of VEN, and its metabolites in biological samples, too. Being a colorimetric estimation, the method can serve as a prototype for the development of analytical method for more complex samples like plasma or serum and can be easily adapted to other sensitive analytical instruments, without much additional rework.

## Figures and Tables

**Table 1:** Regression parameters and derived limits and coefficients of the proposed analytical method.

Parameter	VEN	(NDV + Cyclic VEN)
λ_Max_, nm	608.5	616.5 nm
Beer’s Law range, µg/mL	10–100	5–100
Molar extinction coefficient (l mol^−1^cm^−1^)	0.61 × 10^5^	0.94 × 10^5^
Limit of detection, µg/mL	1.38	1.13
Limit of quantification, µg/mL	3.4	4.1
Sandell sensitivity	0.1638	0.2533
Regression Equation *		
Intercept	−0.0035	0.0109
Slope	0.0097	0.0080
S_a_	0.01235	0.01297
S_b_	0.00014	0.00016
Correlation Coefficient	0.99958	0.99933

* Y = a + bX, where Y is absorbance and X is the concentration (µg/mL); S_a_ = standard deviation of intercept, and S_b_ = standard deviation of the slope.

**Table 2:** Recovery studies of VEN and NDV + Cyclic VEN.

Intra-Day Assessment							
VEN Added (µg/mL)	VEN Obtained (µg/mL)	% RE	% RSD	(NDV + Cyclic VEN) Added (µg/mL)	(NDV + Cyclic VEN) Obtained (µg/mL)	% RE	% RSD
2.0	1.980	1.00	2.9141	2.0	1.990	0.50	2.8994
4.0	3.950	1.25	1.7877	4.0	3.970	0.75	1.7787
6.0	5.930	1.17	0.5029	6.0	5.940	1.00	0.5020
**Inter-Day Assessment**							
**VEN added (µg/mL)**	**VEN obtained** **(µg/mL)**	**% RE**	**% RSD**	**(NDV + Cyclic VEN) added** **(µg/mL)**	**(NDV + Cyclic VEN) obtained** **(µg/mL)**	**% RE**	**% RSD**
2.0	1.970	1.50	2.9288	2.0	1.980	1.00	2.9141
4.0	3.940	1.50	1.7923	4.0	3.960	1.00	1.7832
6.0	5.920	1.33	0.5037	6.0	5.920	1.33	0.5037

**Table 3:** Effect of common excipients—recovery study.

Sl. No.	Excipient	Quantity of Excipient (µg/mL) ^1^	Recovery ^2^ %
1	Talc	100	99.87 ± 0.85
2	Starch	150	99.74 ± 0.82
3	Cellulose	200	99.8 ± 0.78
4	Alginate	100	99.3 ± 0.96
5	Gum Arabic	100	100.2 ± 0.55
6	Lactose	100	99.6 ± 0.85
7	Dextrose	100	99.8 ± 0.77

^1^ VEN concentration used was 10 μg/mL. ^2^ Average of five replicate determinations ± SD.

**Table 4:** Effect of surfactants on the absorbance of 30 µg/mL of VEN.

Sl. No.	Surfactant Added	Amount Added (µg/mL)	Absorbance
1	No surfactant	0	0.61
2	Sodium lauryl sulfate	5	0.62
3	Gelatin	5	0.65
4	Sodium stearate	10	0.60
5	Polysorbate	10	0.60

**Table 5:** Effect of standard addition of mixture of NDV + Cyclic VEN on to VEN samples.

Sl. No.	VEN Taken (µg/mL)	(NDV + Cyclic VEN) Added (µg/mL)	Absorbance
1	20.0	0.0	0.456
2	20.0	0.5	0.462
3	20.0	1.0	0.478
4	20.0	2.0	0.502
5	20.0	4.0	0.546
6	20.0	6.0	0.583
7	20.0	8.0	0.606
8	20.0	10.0	0.655
